# 2-Amino-3,4,5,6-tetra­fluoro­benzoic acid

**DOI:** 10.1107/S1600536811023087

**Published:** 2011-06-18

**Authors:** Xiao-Jian Liao, Wei Guo, Shi-Hai Xu

**Affiliations:** a Department of Chemistry, Jinan University, Guangzhou 510632, People’s Republic of China

## Abstract

The asymmetric unit of the title compound, C_7_H_3_F_4_NO_2_, obtained as an inter­mediate in the synthesis of a coupling reagent, contains four independent and conformationally similar mol­ecules. The amine H atoms form both intra­molecular and inter­molecular N—H⋯O_carbox­yl_ hydrogen bonds which, together with inter­molecular O—H⋯O_carbox­yl_ hydrogen bonds and N—H⋯F associations form ribbon structures along the *a* axis.

## Related literature

The title compound was obtained as one of the inter­mediates in the synthesis of a coupling reagent (Xu *et al.*, 2008 ▶; Liao *et al.*, 2007 ▶), using the Hofmann rearrangement (Perumal & Muthialu, 2004 ▶) with 2-carboxyl-3,4,5,6-tetra­fluoro­benzamide (Cai *et al.*, 1992 ▶).
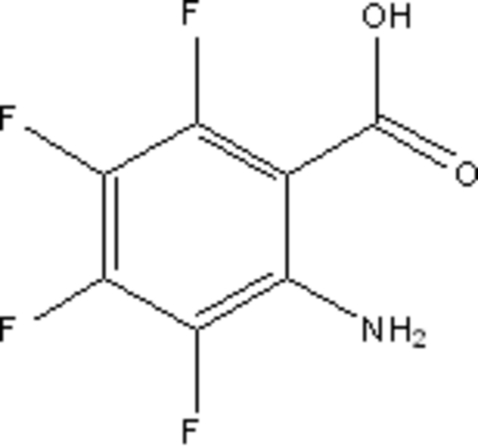


## Experimental

### 

#### Crystal data

C_7_H_3_F_4_NO_2_
*M* *_r_* = 209.10
Triclinic, 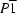

*a* = 11.0367 (11) Å
*b* = 11.3664 (11) Å
*c* = 12.5702 (12) Å
α = 80.378 (8)°
β = 79.764 (8)°
γ = 82.011 (8)°
*V* = 1520.2 (3) Å^3^
*Z* = 8
Cu *K*α radiation
μ = 1.79 mm^−1^
*T* = 295 K
0.50 × 0.30 × 0.15 mm

#### Data collection

Oxford Diffraction Xcalibur Sapphire3 Gemini ultra CCD diffractometer
Absorption correction: multi-scan (*CrysAlis PRO*; Oxford Diffraction, 2010 ▶) *T*_min_ = 0.392, *T*_max_ = 1.000
8708 measured reflections
4791 independent reflections
3521 reflections with *I* > 2σ(*I*)
*R*_int_ = 0.031

#### Refinement

*R*[*F* ^2^ > 2σ(*F* ^2^)] = 0.057
*wR*(*F* ^2^) = 0.177
*S* = 1.05
4791 reflections
533 parameters
16 restraints
H atoms treated by a mixture of independent and constrained refinement
Δρ_max_ = 0.43 e Å^−3^
Δρ_min_ = −0.24 e Å^−3^

### 

Data collection: *CrysAlis PRO* (Oxford Diffraction, 2010 ▶); cell refinement: *CrysAlis PRO*; data reduction: *CrysAlis PRO*; program(s) used to solve structure: *SHELXS97* (Sheldrick, 2008 ▶); program(s) used to refine structure: *SHELXL97* (Sheldrick, 2008 ▶); molecular graphics: *OLEX2* (Dolomanov *et al.*, 2009 ▶); software used to prepare material for publication: *OLEX2*.

## Supplementary Material

Crystal structure: contains datablock(s) global. DOI: 10.1107/S1600536811023087/zs2117sup1.cif

Supplementary material file. DOI: 10.1107/S1600536811023087/zs2117globalsup2.cml

Additional supplementary materials:  crystallographic information; 3D view; checkCIF report

## Figures and Tables

**Table 1:** Hydrogen-bond geometry (Å, °)

*D*—H⋯*A*	*D*—H	H⋯*A*	*D*⋯*A*	*D*—H⋯*A*
N1—H1*B*⋯O4	0.84 (3)	2.04 (4)	2.637 (4)	128 (3)
N1—H1*B*⋯F5^i^	0.84 (3)	2.40 (3)	3.154 (4)	150 (3)
N2—H2*A*⋯O10^ii^	0.83 (3)	2.58 (3)	3.363 (5)	158 (3)
N2—H2*B*⋯O7	0.85 (3)	2.03 (4)	2.643 (5)	129 (3)
N3—H3*A*⋯O11^iii^	0.85 (3)	2.50 (3)	3.337 (4)	167 (3)
N3—H3*B*⋯O5	0.85 (3)	2.04 (4)	2.654 (4)	129 (3)
N4—H4*B*⋯O1	0.86 (3)	2.01 (3)	2.648 (4)	130 (3)
N4—H4*B*⋯F16^iv^	0.86 (3)	2.48 (3)	3.191 (5)	140 (3)
O9—H9⋯O5^v^	0.82	1.84	2.660 (3)	175
O10—H10⋯O4^vi^	0.82	1.86	2.675 (4)	178
O11—H11⋯O1^vi^	0.82	1.82	2.643 (3)	177

Symmetry codes: (i) 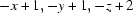; (ii) 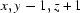; (iii) 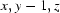; (iv) 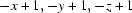; (v) 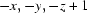; (vi) 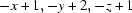.

**Fig. 1.**
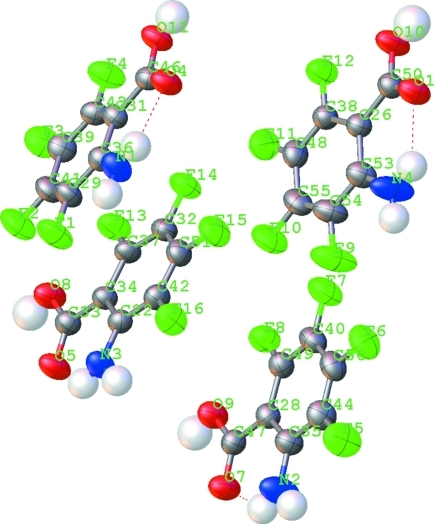
The molecular conformation of the four independent molecules in the asymmetric unit of the title compound, showing the atom numbering scheme. Intramolecular hydrogen bonds are shown as dashed lines. Displacement ellipsoids are drawn at the 50% probability level.

## supplementary crystallographic information

### Comment

The title compound C_7_H_3_F_4_NO_2_ (I) was obtained as one of the intermediates in the synthesis of a coupling reagent (Xu *et al.*, 2008, Liao *et al.*, 2007), using the Hofmann rearrangement (Perumal & Muthialu, 2004) with 2-carboxyl-3,4,5,6-tetrafluorobenzamide (Cai *et al.*, 1992). In the structure of (I), the asymmetric unit contains four independent and conformationally similar molecules (Fig. 1). The molecules associate through carboxylic acid O—H···O_carboxyl_ hydrogen bonds (Table 1) while the amine H atoms form both intramolecular N—H···O_carboxyl_ hydrogen bonds as well as intermolecular N—H···F associations give one-dimensional ribbon structures. Also present in the structure are short intermolecular F···F contacts [minimum, 2.825 (3) Å].

### Experimental

To a stirred solution of 39.2 g of KOH in 356 mL of distilled water, 11.3 g of bromine was added and after 30 min, 70 mmol of 2-carboxy-3,4,5,6-tetrafluorobenzamide was added. After allowing the reaction to proceed for 30 min at 293 K, the mixture was heated to 363K-368K and maintained at that temperature for 4 h, after which the  mixture was cooled to room temperature and allowed to stand for 48 h. To the mixture was then added 100 mL of water, the pH adjusted to 1 at ice-water temperature, stirred and filtered, giving a yellow solid (16.2 g, yield 94%). Pale yellow crystals of (I) suitable for X-ray analysis grew over a period of a week from a solution of the solid in methanol at room temperature.

### Refinement

The carboxylic acid H atoms were positioned geometrically and were included in the refinement in the riding-model approximation with O—H = 0.82 Å and *U*_iso_(H) = 1.5*U*_eq_(O) The amine H atoms were located in difference Fourier maps and the positional parameters were refined but with the displacement parameters riding with *U*_iso_(H) = 1.5*U*_eq_(N).

### Crystal data

**Table:**

C_7_H_3_F_4_NO_2_	*Z* = 8
*M**_r_* = 209.10	*F*(000) = 832
Triclinic, *P*1	*D*_x_ = 1.827 Mg m^−^^3^
*a* = 11.0367 (11) Å	Cu *K*α radiation, λ = 1.5418 Å
*b* = 11.3664 (11) Å	Cell parameters from 4167 reflections
*c* = 12.5702 (12) Å	θ = 3.6–62.7°
α = 80.378 (8)°	µ = 1.79 mm^−^^1^
β = 79.764 (8)°	*T* = 295 K
γ = 82.011 (8)°	Plate, pale yellow
*V* = 1520.2 (3) Å^3^	0.50 × 0.30 × 0.15 mm

### Data collection

**Table:**

Oxford Diffraction Xcalibur Sapphire3 Gemini ultra CCD diffractometer	4791 independent reflections
Radiation source: Enhance Ultra (Cu) X-ray Source	3521 reflections with *I* > 2σ(*I*)
mirror	*R*_int_ = 0.031
Detector resolution: 16.0288 pixels mm^-1^	θ_max_ = 62.8°, θ_min_ = 3.6°
ω scans	*h* = −12→12
Absorption correction: multi-scan (*CrysAlis PRO*; Oxford Diffraction, 2010)	*k* = −12→13
*T*_min_ = 0.392, *T*_max_ = 1.000	*l* = −14→14
8708 measured reflections	

### Refinement

**Table:**

Refinement on *F*^2^	Primary atom site location: structure-invariant direct methods
Least-squares matrix: full	Secondary atom site location: difference Fourier map
*R*[*F*^2^ > 2σ(*F*^2^)] = 0.057	Hydrogen site location: inferred from neighbouring sites
*wR*(*F*^2^) = 0.177	H atoms treated by a mixture of independent and constrained refinement
*S* = 1.05	*w* = 1/[σ^2^(*F*_o_^2^) + (0.0935*P*)^2^ + 0.6844*P*] where *P* = (*F*_o_^2^ + 2*F*_c_^2^)/3
4791 reflections	(Δ/σ)_max_ < 0.001
533 parameters	Δρ_max_ = 0.43 e Å^−^^3^
16 restraints	Δρ_min_ = −0.24 e Å^−^^3^

### Special details

**Table:**

Geometry. All esds (except the esd in the dihedral angle between two l.s. planes) are estimated using the full covariance matrix. The cell esds are taken into account individually in the estimation of esds in distances, angles and torsion angles; correlations between esds in cell parameters are only used when they are defined by crystal symmetry. An approximate (isotropic) treatment of cell esds is used for estimating esds involving l.s. planes.
Refinement. Refinement of F^2^ against ALL reflections. The weighted R-factor wR and goodness of fit S are based on F^2^, conventional R-factors R are based on F, with F set to zero for negative F^2^. The threshold expression of F^2^ > 2sigma(F^2^) is used only for calculating R-factors(gt) etc. and is not relevant to the choice of reflections for refinement. R-factors based on F^2^ are statistically about twice as large as those based on F, and R- factors based on ALL data will be even larger.

### Fractional atomic coordinates and isotropic or equivalent isotropic displacement parameters (Å^2^)

**Table:**

	*x*	*y*	*z*	*U*_iso_*/*U*_eq_	
F16	0.18790 (19)	0.2980 (2)	0.63873 (16)	0.0672 (6)	
F13	−0.0401 (2)	0.42992 (19)	0.28267 (16)	0.0682 (6)	
F14	0.1447 (2)	0.54588 (19)	0.30496 (17)	0.0701 (6)	
O8	−0.1849 (2)	0.2707 (2)	0.3563 (2)	0.0632 (7)	
H8	−0.2349	0.2248	0.3531	0.095*	
F15	0.2604 (2)	0.4812 (2)	0.48248 (19)	0.0747 (6)	
O5	−0.1389 (2)	0.1291 (2)	0.49241 (19)	0.0622 (7)	
C22	0.0365 (3)	0.2621 (3)	0.5411 (2)	0.0441 (7)	
N3	0.0065 (3)	0.1737 (3)	0.6245 (2)	0.0581 (8)	
C32	0.1097 (3)	0.4535 (3)	0.3810 (3)	0.0502 (8)	
C33	−0.1201 (3)	0.2250 (3)	0.4350 (2)	0.0470 (7)	
C34	−0.0239 (3)	0.2952 (3)	0.4488 (2)	0.0443 (7)	
C37	0.0151 (3)	0.3923 (3)	0.3714 (2)	0.0456 (7)	
C42	0.1311 (3)	0.3284 (3)	0.5489 (3)	0.0491 (8)	
C51	0.1684 (3)	0.4208 (3)	0.4715 (3)	0.0524 (8)	
F8	0.3963 (2)	0.21194 (19)	0.48315 (16)	0.0667 (6)	
O9	0.2802 (2)	0.0302 (2)	0.5375 (2)	0.0615 (6)	
H9	0.2341	−0.0183	0.5317	0.092*	
O7	0.3410 (2)	−0.1190 (2)	0.6616 (2)	0.0627 (7)	
F5	0.6708 (2)	0.0512 (2)	0.8060 (2)	0.0892 (8)	
F6	0.6953 (2)	0.2663 (2)	0.6819 (2)	0.0787 (7)	
F7	0.5568 (2)	0.3471 (2)	0.5210 (2)	0.0827 (7)	
C28	0.4414 (3)	0.0572 (3)	0.6287 (2)	0.0462 (7)	
C35	0.5150 (3)	0.0154 (3)	0.7126 (3)	0.0531 (8)	
C40	0.5419 (3)	0.2401 (3)	0.5830 (3)	0.0577 (9)	
C44	0.5992 (3)	0.0896 (3)	0.7262 (3)	0.0601 (9)	
C47	0.3507 (3)	−0.0174 (3)	0.6118 (2)	0.0469 (7)	
C49	0.4586 (3)	0.1696 (3)	0.5659 (3)	0.0498 (8)	
N2	0.5075 (4)	−0.0899 (3)	0.7795 (3)	0.0833 (12)	
C56	0.6129 (3)	0.1984 (3)	0.6649 (3)	0.0583 (9)	
F4	0.0302 (2)	0.94676 (19)	0.75391 (15)	0.0700 (6)	
O11	0.1722 (2)	1.0628 (2)	0.82373 (17)	0.0570 (6)	
H11	0.2109	1.1185	0.8268	0.085*	
O4	0.2335 (2)	0.9962 (2)	0.98460 (19)	0.0594 (6)	
F3	−0.1220 (2)	0.7811 (2)	0.78819 (17)	0.0745 (6)	
F2	−0.1394 (2)	0.6232 (2)	0.97776 (19)	0.0815 (7)	
F1	−0.0109 (2)	0.6443 (2)	1.13507 (17)	0.0810 (7)	
N1	0.1387 (3)	0.8133 (3)	1.1147 (2)	0.0620 (8)	
C29	0.0014 (3)	0.7205 (3)	1.0410 (3)	0.0543 (8)	
C31	0.0926 (3)	0.8903 (3)	0.9301 (2)	0.0432 (7)	
C36	0.0799 (3)	0.8091 (3)	1.0291 (2)	0.0471 (7)	
C39	−0.0549 (3)	0.7890 (3)	0.8658 (3)	0.0544 (8)	
C41	−0.0645 (3)	0.7095 (3)	0.9618 (3)	0.0565 (9)	
C43	0.0231 (3)	0.8750 (3)	0.8509 (2)	0.0473 (7)	
C46	0.1720 (3)	0.9855 (3)	0.9152 (2)	0.0464 (7)	
F12	0.4204 (2)	0.75684 (19)	−0.01077 (18)	0.0705 (6)	
F11	0.2839 (2)	0.5761 (2)	0.0206 (2)	0.0766 (7)	
O10	0.6074 (3)	0.8442 (2)	0.0225 (2)	0.0687 (7)	
H10	0.6552	0.8943	0.0195	0.103*	
O1	0.6965 (2)	0.7627 (2)	0.1668 (2)	0.0632 (7)	
C26	0.5475 (3)	0.6595 (3)	0.1206 (2)	0.0451 (7)	
F10	0.3353 (2)	0.3778 (2)	0.1668 (2)	0.0847 (7)	
F9	0.5247 (3)	0.3629 (2)	0.2782 (2)	0.1045 (10)	
C38	0.4484 (3)	0.6618 (3)	0.0631 (3)	0.0487 (8)	
N4	0.6652 (4)	0.5390 (3)	0.2570 (3)	0.0928 (14)	
C48	0.3770 (3)	0.5711 (3)	0.0787 (3)	0.0544 (8)	
C50	0.6226 (3)	0.7589 (3)	0.1051 (3)	0.0497 (8)	
C53	0.5726 (4)	0.5559 (3)	0.1963 (3)	0.0582 (9)	
C54	0.4979 (4)	0.4638 (3)	0.2087 (3)	0.0660 (10)	
C55	0.4029 (4)	0.4695 (3)	0.1527 (3)	0.0597 (9)	
H3A	0.059 (3)	0.147 (3)	0.667 (2)	0.072*	
H3B	−0.039 (3)	0.123 (3)	0.615 (3)	0.072*	
H2A	0.551 (3)	−0.113 (3)	0.828 (2)	0.100*	
H2B	0.463 (3)	−0.140 (2)	0.768 (3)	0.100*	
H1A	0.129 (3)	0.760 (3)	1.1720 (19)	0.074*	
H1B	0.186 (3)	0.866 (3)	1.112 (3)	0.074*	
H4A	0.681 (3)	0.476 (2)	0.299 (3)	0.107*	
H4B	0.713 (3)	0.595 (2)	0.249 (3)	0.107*	

### Atomic displacement parameters (Å^2^)

**Table:**

	*U*^11^	*U*^22^	*U*^33^	*U*^12^	*U*^13^	*U*^23^
F16	0.0711 (13)	0.0830 (14)	0.0560 (11)	−0.0196 (11)	−0.0327 (10)	−0.0008 (10)
F13	0.0819 (14)	0.0688 (13)	0.0578 (11)	−0.0236 (11)	−0.0350 (10)	0.0176 (10)
F14	0.0769 (14)	0.0623 (13)	0.0693 (13)	−0.0277 (11)	−0.0150 (11)	0.0155 (10)
O8	0.0711 (16)	0.0587 (15)	0.0679 (15)	−0.0207 (12)	−0.0400 (13)	0.0094 (12)
F15	0.0702 (14)	0.0751 (14)	0.0891 (15)	−0.0329 (11)	−0.0289 (11)	−0.0035 (12)
O5	0.0732 (16)	0.0615 (15)	0.0586 (14)	−0.0300 (13)	−0.0300 (12)	0.0119 (12)
C22	0.0460 (17)	0.0459 (17)	0.0392 (15)	−0.0039 (14)	−0.0091 (13)	−0.0018 (13)
N3	0.0614 (18)	0.0679 (19)	0.0467 (15)	−0.0201 (15)	−0.0227 (13)	0.0121 (14)
C32	0.0527 (19)	0.0426 (17)	0.0533 (18)	−0.0106 (15)	−0.0077 (15)	0.0028 (15)
C33	0.0510 (18)	0.0487 (18)	0.0421 (16)	−0.0080 (15)	−0.0120 (14)	−0.0019 (14)
C34	0.0477 (17)	0.0444 (17)	0.0414 (15)	−0.0077 (14)	−0.0125 (13)	0.0001 (13)
C37	0.0526 (18)	0.0442 (17)	0.0402 (16)	−0.0077 (14)	−0.0163 (13)	0.0046 (13)
C42	0.0504 (18)	0.0532 (19)	0.0455 (16)	−0.0057 (15)	−0.0171 (14)	−0.0023 (15)
C51	0.0479 (18)	0.055 (2)	0.0574 (19)	−0.0131 (15)	−0.0134 (15)	−0.0069 (16)
F8	0.0722 (13)	0.0644 (13)	0.0647 (12)	−0.0157 (10)	−0.0253 (10)	0.0096 (10)
O9	0.0639 (15)	0.0617 (15)	0.0664 (15)	−0.0199 (12)	−0.0318 (12)	0.0028 (12)
O7	0.0704 (16)	0.0519 (14)	0.0726 (15)	−0.0180 (12)	−0.0341 (13)	0.0049 (12)
F5	0.0981 (18)	0.0973 (18)	0.0898 (16)	−0.0335 (14)	−0.0588 (14)	0.0020 (14)
F6	0.0734 (14)	0.0865 (16)	0.0894 (15)	−0.0374 (12)	−0.0187 (12)	−0.0206 (13)
F7	0.0925 (17)	0.0697 (14)	0.0882 (16)	−0.0367 (13)	−0.0184 (13)	0.0091 (12)
C28	0.0464 (17)	0.0485 (18)	0.0459 (17)	−0.0093 (14)	−0.0086 (14)	−0.0083 (14)
C35	0.059 (2)	0.054 (2)	0.0506 (18)	−0.0128 (16)	−0.0177 (15)	−0.0036 (15)
C40	0.062 (2)	0.052 (2)	0.060 (2)	−0.0190 (17)	−0.0034 (17)	−0.0053 (16)
C44	0.064 (2)	0.068 (2)	0.056 (2)	−0.0163 (19)	−0.0233 (17)	−0.0101 (18)
C47	0.0461 (17)	0.0507 (19)	0.0457 (16)	−0.0047 (14)	−0.0126 (13)	−0.0069 (15)
C49	0.0493 (18)	0.056 (2)	0.0443 (16)	−0.0094 (15)	−0.0093 (14)	−0.0033 (15)
N2	0.108 (3)	0.072 (2)	0.084 (2)	−0.037 (2)	−0.061 (2)	0.0190 (19)
C56	0.055 (2)	0.066 (2)	0.060 (2)	−0.0208 (17)	−0.0073 (16)	−0.0166 (18)
F4	0.1041 (17)	0.0657 (13)	0.0480 (11)	−0.0320 (12)	−0.0316 (11)	0.0086 (9)
O11	0.0755 (16)	0.0548 (14)	0.0449 (12)	−0.0271 (12)	−0.0167 (11)	0.0045 (10)
O4	0.0715 (16)	0.0568 (14)	0.0564 (13)	−0.0259 (12)	−0.0274 (12)	0.0074 (11)
F3	0.0953 (16)	0.0776 (14)	0.0655 (13)	−0.0313 (12)	−0.0364 (12)	−0.0099 (11)
F2	0.0936 (17)	0.0807 (15)	0.0808 (15)	−0.0517 (13)	−0.0238 (12)	0.0036 (12)
F1	0.0971 (17)	0.0848 (16)	0.0630 (13)	−0.0440 (13)	−0.0275 (12)	0.0279 (12)
N1	0.069 (2)	0.077 (2)	0.0442 (15)	−0.0264 (16)	−0.0210 (14)	0.0075 (15)
C29	0.059 (2)	0.058 (2)	0.0457 (17)	−0.0195 (17)	−0.0085 (15)	0.0060 (15)
C31	0.0484 (17)	0.0452 (17)	0.0376 (15)	−0.0105 (14)	−0.0079 (13)	−0.0051 (13)
C36	0.0507 (18)	0.0489 (18)	0.0426 (16)	−0.0084 (15)	−0.0100 (13)	−0.0043 (14)
C39	0.063 (2)	0.060 (2)	0.0478 (18)	−0.0139 (17)	−0.0203 (15)	−0.0117 (16)
C41	0.061 (2)	0.053 (2)	0.059 (2)	−0.0229 (17)	−0.0120 (16)	−0.0037 (16)
C43	0.061 (2)	0.0457 (17)	0.0366 (15)	−0.0092 (15)	−0.0140 (14)	−0.0014 (13)
C46	0.0519 (18)	0.0437 (17)	0.0436 (16)	−0.0070 (14)	−0.0087 (14)	−0.0043 (14)
F12	0.0690 (13)	0.0618 (13)	0.0836 (14)	−0.0162 (10)	−0.0357 (11)	0.0138 (11)
F11	0.0623 (13)	0.0722 (14)	0.1048 (17)	−0.0188 (11)	−0.0332 (12)	−0.0095 (13)
O10	0.0822 (18)	0.0604 (15)	0.0701 (16)	−0.0318 (13)	−0.0379 (14)	0.0190 (13)
O1	0.0750 (16)	0.0601 (15)	0.0614 (14)	−0.0257 (13)	−0.0317 (13)	0.0084 (12)
C26	0.0489 (17)	0.0449 (17)	0.0435 (16)	−0.0083 (14)	−0.0119 (13)	−0.0044 (14)
F10	0.0967 (18)	0.0647 (14)	0.1022 (17)	−0.0426 (13)	−0.0251 (14)	−0.0014 (13)
F9	0.155 (3)	0.0682 (15)	0.1023 (19)	−0.0487 (17)	−0.0663 (18)	0.0320 (14)
C38	0.0496 (18)	0.0476 (18)	0.0487 (17)	−0.0077 (15)	−0.0109 (14)	−0.0010 (14)
N4	0.129 (3)	0.061 (2)	0.105 (3)	−0.040 (2)	−0.079 (3)	0.028 (2)
C48	0.0472 (19)	0.059 (2)	0.062 (2)	−0.0116 (16)	−0.0123 (15)	−0.0159 (17)
C50	0.0506 (18)	0.0513 (19)	0.0488 (17)	−0.0112 (15)	−0.0113 (14)	−0.0038 (15)
C53	0.070 (2)	0.056 (2)	0.0538 (19)	−0.0184 (18)	−0.0208 (17)	−0.0015 (16)
C54	0.094 (3)	0.049 (2)	0.058 (2)	−0.023 (2)	−0.0221 (19)	0.0061 (17)
C55	0.068 (2)	0.051 (2)	0.064 (2)	−0.0235 (17)	−0.0069 (18)	−0.0102 (17)

### Geometric parameters (Å, °)

**Table:**

F16—C42	1.360 (3)	F4—C43	1.345 (3)
F13—C37	1.344 (3)	O11—H11	0.8200
F14—C32	1.346 (4)	O11—C46	1.325 (4)
O8—H8	0.8200	O4—C46	1.227 (4)
O8—C33	1.318 (4)	F3—C39	1.346 (4)
F15—C51	1.341 (4)	F2—C41	1.337 (4)
O5—C33	1.224 (4)	F1—C29	1.341 (4)
C22—N3	1.354 (4)	N1—C36	1.362 (4)
C22—C34	1.412 (4)	N1—H1A	0.859 (17)
C22—C42	1.396 (5)	N1—H1B	0.846 (17)
N3—H3A	0.855 (17)	C29—C36	1.390 (5)
N3—H3B	0.854 (17)	C29—C41	1.364 (5)
C32—C37	1.365 (5)	C31—C36	1.417 (4)
C32—C51	1.381 (5)	C31—C43	1.406 (4)
C33—C34	1.463 (4)	C31—C46	1.454 (4)
C34—C37	1.405 (4)	C39—C41	1.379 (5)
C42—C51	1.361 (5)	C39—C43	1.360 (5)
F8—C49	1.335 (4)	F12—C38	1.341 (4)
O9—H9	0.8200	F11—C48	1.353 (4)
O9—C47	1.320 (4)	O10—H10	0.8200
O7—C47	1.228 (4)	O10—C50	1.314 (4)
F5—C44	1.363 (4)	O1—C50	1.229 (4)
F6—C56	1.337 (4)	C26—C38	1.410 (5)
F7—C40	1.344 (4)	C26—C50	1.460 (4)
C28—C35	1.424 (4)	C26—C53	1.413 (5)
C28—C47	1.462 (4)	F10—C55	1.334 (4)
C28—C49	1.403 (5)	F9—C54	1.353 (4)
C35—C44	1.388 (5)	C38—C48	1.351 (5)
C35—N2	1.347 (5)	N4—C53	1.356 (5)
C40—C49	1.367 (5)	N4—H4A	0.827 (17)
C40—C56	1.385 (5)	N4—H4B	0.869 (17)
C44—C56	1.356 (5)	C48—C55	1.388 (5)
N2—H2A	0.829 (17)	C53—C54	1.393 (5)
N2—H2B	0.851 (17)	C54—C55	1.352 (5)
			
C33—O8—H8	109.5	C46—O11—H11	109.5
N3—C22—C34	124.9 (3)	C36—N1—H1A	120 (2)
N3—C22—C42	117.6 (3)	C36—N1—H1B	121 (2)
C42—C22—C34	117.4 (3)	H1A—N1—H1B	119 (3)
C22—N3—H3A	118 (2)	F1—C29—C36	118.3 (3)
C22—N3—H3B	119 (2)	F1—C29—C41	119.1 (3)
H3A—N3—H3B	115 (3)	C41—C29—C36	122.6 (3)
F14—C32—C37	121.1 (3)	C36—C31—C46	120.2 (3)
F14—C32—C51	119.9 (3)	C43—C31—C36	116.8 (3)
C37—C32—C51	119.0 (3)	C43—C31—C46	123.0 (3)
O8—C33—C34	116.0 (3)	N1—C36—C29	117.5 (3)
O5—C33—O8	121.8 (3)	N1—C36—C31	124.0 (3)
O5—C33—C34	122.2 (3)	C29—C36—C31	118.5 (3)
C22—C34—C33	119.3 (3)	F3—C39—C41	120.3 (3)
C37—C34—C22	117.9 (3)	F3—C39—C43	120.7 (3)
C37—C34—C33	122.7 (3)	C43—C39—C41	119.0 (3)
F13—C37—C32	116.2 (3)	F2—C41—C29	120.2 (3)
F13—C37—C34	121.0 (3)	F2—C41—C39	120.1 (3)
C32—C37—C34	122.8 (3)	C29—C41—C39	119.7 (3)
F16—C42—C22	117.5 (3)	F4—C43—C31	121.1 (3)
F16—C42—C51	119.2 (3)	F4—C43—C39	115.5 (3)
C51—C42—C22	123.3 (3)	C39—C43—C31	123.4 (3)
F15—C51—C32	119.5 (3)	O11—C46—C31	116.6 (3)
F15—C51—C42	121.0 (3)	O4—C46—O11	121.3 (3)
C42—C51—C32	119.5 (3)	O4—C46—C31	122.0 (3)
C47—O9—H9	109.5	C50—O10—H10	109.5
C35—C28—C47	119.2 (3)	C38—C26—C50	122.7 (3)
C49—C28—C35	118.2 (3)	C38—C26—C53	117.4 (3)
C49—C28—C47	122.6 (3)	C53—C26—C50	119.9 (3)
C44—C35—C28	117.2 (3)	F12—C38—C26	120.4 (3)
N2—C35—C28	124.5 (3)	F12—C38—C48	116.6 (3)
N2—C35—C44	118.3 (3)	C48—C38—C26	123.0 (3)
F7—C40—C49	121.2 (3)	C53—N4—H4A	123 (2)
F7—C40—C56	120.2 (3)	C53—N4—H4B	118 (2)
C49—C40—C56	118.6 (3)	H4A—N4—H4B	118 (3)
F5—C44—C35	118.1 (3)	F11—C48—C55	119.7 (3)
C56—C44—F5	118.6 (3)	C38—C48—F11	121.1 (3)
C56—C44—C35	123.3 (3)	C38—C48—C55	119.2 (3)
O9—C47—C28	116.0 (3)	O10—C50—C26	116.9 (3)
O7—C47—O9	121.3 (3)	O1—C50—O10	121.6 (3)
O7—C47—C28	122.8 (3)	O1—C50—C26	121.5 (3)
F8—C49—C28	121.3 (3)	N4—C53—C26	124.7 (3)
F8—C49—C40	116.1 (3)	N4—C53—C54	117.5 (3)
C40—C49—C28	122.6 (3)	C54—C53—C26	117.8 (3)
C35—N2—H2A	123 (2)	F9—C54—C53	117.9 (3)
C35—N2—H2B	120 (2)	C55—C54—F9	118.9 (3)
H2A—N2—H2B	117 (3)	C55—C54—C53	123.2 (3)
F6—C56—C40	119.2 (3)	F10—C55—C48	119.9 (3)
F6—C56—C44	120.7 (3)	F10—C55—C54	120.7 (3)
C44—C56—C40	120.2 (3)	C54—C55—C48	119.4 (3)

### Hydrogen-bond geometry (Å, °)

**Table:**

*D*—H···*A*	*D*—H	H···*A*	*D*···*A*	*D*—H···*A*
N1—H1A···F1	0.86 (3)	2.31 (3)	2.655 (4)	104 (2)
N1—H1B···O4	0.84 (3)	2.04 (4)	2.637 (4)	128 (3)
N1—H1B···F5^i^	0.84 (3)	2.40 (3)	3.154 (4)	150 (3)
N2—H2A···F5	0.83 (3)	2.39 (3)	2.670 (5)	101 (2)
N2—H2A···O10^ii^	0.83 (3)	2.58 (3)	3.363 (5)	158 (3)
N2—H2B···O7	0.85 (3)	2.03 (4)	2.643 (5)	129 (3)
N3—H3A···F4^iii^	0.85 (3)	2.39 (3)	2.816 (4)	112 (3)
N3—H3A···F16	0.85 (3)	2.32 (3)	2.652 (4)	104 (2)
N3—H3A···O11^iii^	0.85 (3)	2.50 (3)	3.337 (4)	167 (3)
N3—H3B···O5	0.85 (3)	2.04 (4)	2.654 (4)	129 (3)
N4—H4A···F9	0.83 (3)	2.36 (3)	2.650 (5)	101 (3)
N4—H4B···O1	0.86 (3)	2.01 (3)	2.648 (4)	130 (3)
N4—H4B···F16^iv^	0.86 (3)	2.48 (3)	3.191 (5)	140 (3)
O9—H9···O5^v^	0.82	1.84	2.660 (3)	175
O10—H10···O4^vi^	0.82	1.86	2.675 (4)	178
O11—H11···O1^vi^	0.82	1.82	2.643 (3)	177

Symmetry codes: (i) −*x*+1, −*y*+1, −*z*+2; (ii) *x*, *y*−1, *z*+1; (iii) *x*, *y*−1, *z*; (iv) −*x*+1, −*y*+1, −*z*+1; (v) −*x*, −*y*, −*z*+1; (vi) −*x*+1, −*y*+2, −*z*+1.
